# Longitudinal Change of Mental Health among Active Social Media Users in China during the COVID-19 Outbreak

**DOI:** 10.3390/healthcare9070833

**Published:** 2021-07-01

**Authors:** Tianli Liu, Sijia Li, Xiaochun Qiao, Xinming Song

**Affiliations:** 1 Institute of Population Research, Peking University, Beijing 100871, China; qxc@pku.edu.cn (X.Q.); xmsong@pku.edu.cn (X.S.); 2 Institute of Psychology, Chinese Academy of Sciences, Beijing 100101, China; lisj@psych.ac.cn; 3 Department of Psychology, University of Chinese Academy of Sciences, Beijing 100049, China

**Keywords:** mental health, COVID-19, SCL-90, longitudinal analysis, computerized machine learning models, Sina Weibo

## Abstract

During the COVID-19 pandemic, every day, updated case numbers and the lasting time of the pandemic became major concerns of people. We collected the online data (28 January to 7 March 2020 during the COVID-19 outbreak) of 16,453 social media users living in mainland China. Computerized machine learning models were developed to estimate their daily scores of the nine dimensions of the Symptom Checklist—90 (SCL-90). Repeated measures analysis of variance (ANOVA) was used to compare the SCL-90 dimension scores between Wuhan and non-Wuhan residents. Fixed effect models were used to analyze the relation of the estimated SCL-90 scores with the daily reported cumulative case numbers and lasting time of the epidemic among Wuhan and non-Wuhan users. In non-Wuhan users, the estimated scores for all the SCL-90 dimensions significantly increased with the lasting time of the epidemic and the accumulation of cases, except for the interpersonal sensitivity dimension. In Wuhan users, although the estimated scores for all nine SCL-90 dimensions significantly increased with the cumulative case numbers, the magnitude of the changes was generally smaller than that in non-Wuhan users. The mental health of Chinese Weibo users was affected by the daily updated information on case numbers and the lasting time of the COVID-19 outbreak.

## 1. Introduction

Since December 2019, when COVID-19 was first reported in Wuhan, China, the highly contagious disease has rapidly spread all over the world [1]. Although the virus severely attacked human beings’ respiratory systems [2], people’s mental health was unavoidably affected due to the unknown and contagious nature of the virus [3], the long length of the epidemic [4] and city lockdowns and travel constrain policies [5], among other factors. In fact, the number of people who reported psychological concerns is much larger than the number of people who were physically affected by the virus [6].

The development of the epidemic caught everyone’s attention. Every day, the first thing most people did was browse the news and check the updated case numbers. A previous cross-sectional study found that a small number of COVID-19 confirmed cases already had an impact on mental health indicators [7], resulting in psychological distress. Therefore, it is essential to understand the potential psychological consequences caused by the daily increasing number of cases.

Meanwhile, a substantial amount of studies in different countries reported a variety of mental health problems, including stress, insomnia, somatization, panic, posttraumatic stress disorder, anxiety and depression, among health workers [8,9] as well as among the general population [10]. Health workers were at heightened risk of mental health problems (e.g., depression and anxiety) in the short term [11]. People’s negative emotions were correlated with the severity of the epidemic [12]. As we know, the majority of these studies collected data at one or two points in time, hence explaining the lack of longitudinal observations of the development of mental health problems among people.

The situation of the epidemic changed very quickly [13]. In December 2019, COVID-19 was first reported in Wuhan, China [14]. On 20 January 2020, Wuhan was locked down in order to slow down the spread of the epidemic [15]. Nevertheless, the virus rapidly arrived in other parts of China [16]. From late January to the middle of February, the number of daily diagnosed cases continuously increased, and the number of new diagnosed cases peaked in the middle of February. Then, the epidemic was mitigated. The number of newly diagnosed cases gradually declined. 20 February was the turning point of the epidemic, when the cumulative number of cured and discharged cases exceeded the number of newly diagnosed cases in both Wuhan and non-Wuhan areas. In March, the newly diagnosed cases number continuously decreased, and the epidemic was effectively controlled in China. We wonder how people’s nerves tightened and eased over the course of the epidemic.

As we know, researchers rarely collect daily data on a population’s mental health, since it is extremely difficult to ask study participants to repeatedly complete the same set of questionnaires every day. In recent years, artificial intelligence has made real-time monitoring of individuals’ psychological statuses possible [17]. It utilizes social media data, which was posted every day, to estimate the users’ psychological statuses.

In the current study, we utilized computerized machine learning models to estimate people’s daily mental health statuses during the study period and to investigate how people’s mental health changed with the development of the epidemic. This study has three specific objectives: (1) to compare the mental health between Wuhan and non-Wuhan social media users; (2) to estimate the relation between the daily updated case numbers and the mental health among Wuhan and non-Wuhan social media users; and (3) to estimate the relation between the lasting time of the pandemic and the mental health among Wuhan and non-Wuhan social media users.

## 2. Methods

### 2.1. Study Sample

This is a longitudinal study to estimate impact of the COVID-19 outbreak on the mental health of people living in Wuhan (the site of the first reported cases) and non-Wuhan areas in mainland China. The samples were selected from an original Sina Weibo (the largest microblog platform in China, similar to Twitter in the US) pool that contained more than 1.16 million Weibo users [18]. In order to collect sufficient information and to ensure the accuracy of the prediction scores, we employed several criteria to select the study sample (Table 1). The inclusion criteria were as follows: (1) Weibo users wrote at least 50 original Weibo posts from 31 December 2019 to 26 January 2020. We used this criteria to recruit active Weibo users who could provide sufficient longitudinal data during the study period; (2) User who were individuals and not institutions; and (3) users whose regional authentication was in mainland China and not “overseas” or “other”. In all, 16,453 Weibo users registered in 432 cities met the inclusion criteria and were included in the current study. Their online data (e.g., posts and records of online behaviors) from 28 January 2020 to 7 March 2020 were analyzed. Every user had data on the estimated SCL-90 scores for each of the 40 days during the study period. The characteristics of the sample were identified according to their registration profile information at Sina Weibo. Among the 16,453 users, 11,961 (72.7%) were female, 390 (2.37%) and were registered as living in Wuhan, the site of the first cases in China. Ages were reported by 3742 (22.74%) users, and among them, 3308 (88.4%) were young people in their 20s or 30s. There was no significant difference in age or sex between the Wuhan and non-Wuhan users. The study’s ethics code is H15009, and it was approved by the IRB at the Institute of Psychology at the Chinese Academy of Sciences.

### 2.2. Machine Learning Model to Estimate SCL-90 Mental Health

Machine learning models were developed by the Computational Cyber-Psychology Lab of the Chinese Academy of Sciences (http://ccpl.psych.ac.cn, accessed on 30 June 2021). Briefly, in 2012, the model development study [19] recruited 563 social media users and asked them to complete the SCL-90 questionnaire [20]. Meanwhile, data were collected on their online behavior characteristics and linguistic features. The SCL-90 questionnaire has been widely used to screen symptoms of mental health disorders among a general population [21]. The nine dimensions of the SCL-90 questionnaire include somatization, obsessive-compulsive disorder, interpersonal sensitivity, depression, anxiety, hostility, phobic anxiety, paranoid ideation and psychoticism. Intensive previous research tested the psychometric properties of the questionnaire and reported very good reliability and validity [21]. By utilizing the questionnaire scores as the gold standard, researchers employed machine learning methods to identify online features that were relevant to the concept of the SCL-90 dimensions and to develop models to predict the mental health level that each SCL-90 dimension concerned. The model estimates the dimensions’ scores based on online behavior characteristics and linguistic features over the past 7 days. Validation studies showed that the estimated scores and the actual questionnaire scores were moderately correlated, and the Pearson correlation coefficients ranged from 0.49 to 0.65 [22]. The major advantage of this method is that people’s mental health can be monitored in a timely manner without asking the subjects to repeatedly complete the same set of questionnaires.

### 2.3. Numbers of Cumulative Cases

Starting from 28 January 2020, the National Health Commission of the People’s Republic of China (http://www.nhc.gov.cn/xcs/yqtb/list_gzbd.shtml, accessed on 1 July 2021) and the health commissions of the local governments (e.g., the Health Commission of Wuhan: http://wjw.wuhan.gov.cn/gsgg/, accessed on 1 July 2021) began to officially report the numbers of new and cumulative cases as well as numbers of deaths every day. The daily reported numbers of the cumulative cases referred to the total number of cases that were diagnosed up to that day, which was calculated based on the number of confirmed cases reported daily by the health commissions. At the end of the study period, the number of cumulative cases for the Wuhan area was 49,871, much higher than the other cities, where the number ranged from 0 to 3518.

### 2.4. Statistical Analysis

Time series plots presented the daily average of the estimated SCL-90 dimension scores in Wuhan and non-Wuhan residents. Repeated measures analysis of variance (ANOVA) was used to compare the SCL-90 dimension scores between Wuhan and non-Wuhan residents. The average of the SCL-90 dimensions’ scores through the study period, which were the means of the 39 daily average scores, were compared with the population norms [23]. Fixed effect models [24] were used to quantify the impact of the COVID-19 epidemic on people’s mental health. The fixed effect model has been widely used in longitudinal analysis to quantify the effect of time-varying covariates. It is the same as a pre-post t-test when the data were collected at two time points. For Wuhan users, nine separate fixed effect models were built, with the nine estimated SCL-90 dimension scores as the dependent variables. Similarly, another nine such models were built for non-Wuhan users. The independent variables included time, daily updated cumulative case numbers and two interaction terms. One was the interaction between sex and time, and the other was the interaction between the epidemic turning point and time. The epidemic turning point was a binary variable coded as 0 if the time was before 20 February and 1 if after 20 February. All statistical analyses were conducted by SAS 9.4 software (SAS Institute, Inc., Cary, NC, USA).

## 3. Results

### 3.1. Comparison of Mental Health between Wuhan and Non-Wuhan Weibo Users

**Figure 1:**
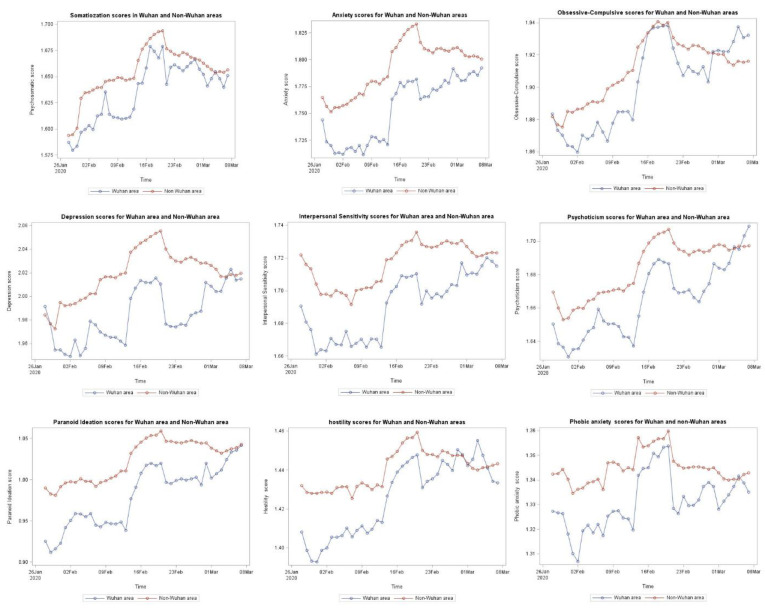
Estimated average daily scores for the nine dimensions of the SCL-90 among Wuhan and non-Wuhan users.

Figure 1 illustrates the longitudinal change of the estimated SCL-90 scores among Wuhan and non-Wuhan Weibo users. From 28 January to 20 February, the curves for all nine dimensions of the SCL-90 mental health scores for the Wuhan users were generally below the curves for the non-Wuhan users. In some of the days between 20 February and 8 March, the curves for five SCL-90 dimensions for Wuhan users, including obsessive-compulsive disorder, depression, psychoticism, hostility and phobic anxiety, reached above the curves for the non-Wuhan users on some days.

Table 2 lists the means and standard deviations for the estimated SCL-90 dimension scores in the selected 5 days, as well as the results of the repeated measures ANOVA that analyzed every day’s data from 28 January to 7 March (40 days in total) to compare the mental health levels between Wuhan and non-Wuhan users. The results of the repeated measures ANOVA found that the Wuhan and non-Wuhan users were significantly different in the SCL-90 dimensions of anxiety (*F* = 5.13, *p* = 0.0235), interpersonal sensitivity (*F* = 6.31, *p* = 0.012), paranoid ideation (*F* = 9.00, *p* = 0.0027), hostility (*F* = 3.94, *p* = 0.0473) and phobic anxiety (*F* = 4.60, *p* = 0.032). Although the repeated measures ANOVA tests offered no direction per se as to which group was more severely affected by mental health problems, the descriptive analysis results (mean and series plots) showed that, in comparison with the non-Wuhan users, the Wuhan users on average scored lower in all five of these dimensions.

Table 3 compares the average of the 40 days’ estimated SCL-90 scores with the population norms. The average scores for the seven dimensions of somatization, anxiety, obsessive-compulsive disorder, interpersonal sensitivity, depression, psychoticism and phobic anxiety, were significantly higher in the Wuhan and non-Wuhan users than the population norms, while the average scores for the other two dimensions, paranoid ideation and hostility, were significantly lower than the population norms.

Table 4 lists the results for 18 separate fixed effect models that were built for each SCL-90 dimension in the Wuhan and non-Wuhan users separately.

### 3.2. Number of Cumulative Cases and SCL-90 Mental Health

The accumulation of COVID-19 cases significantly affected the mental health of both Wuhan and Non-Wuhan users. Almost all of the 18 models with SCL-90-dimension scores as the dependent variables showed that the level of mental health problems increased with the accumulation of COVID-19 cases, except for the model for the psychoticism dimension among Wuhan users, which was marginally significant (*p* = 0.0572), and for the model for the interpersonal sensitivity dimension among non-Wuhan users, which was not significant (*p* = 0.6843). During the study period, generally, Weibo users’ mental health deteriorated with the increase of the cumulative case number.

### 3.3. Time and SCL-90 Mental Health

For non-Wuhan users, the level of mental health problems measured by all nine SCL-90 dimensions significantly increased over time during the epidemic. After 20 February, the increasing rate slightly slowed down, as estimated by the interactions between the time and the turning point (20 February). For instance, before 20 February, for every day the epidemic extended longer, the estimated anxiety score increased by around 0.00217 (95% CI: 0.00207, 0.00227), while the daily increasing rate slightly decreased (*β* = −0.06934 × 10^−5^, 95% CI: −0.07900 × 10^−5^, −0.05968 × 10^−5^) after 20 February. There was one dimension, interpersonal sensitivity, in which the score’s increasing rate slightly accelerated (*β* = 0.03987 × 10^−5^, 95% CI: 0.03230 × 10^−5^, 0.04744 × 10^−5^) after 20 February.

For Wuhan users, the estimated scores for six of the SCL-90 dimensions did not show significant change over time. Scores for two of the dimensions, namely psychoticism (*β* = 0.00135, 95% CI: 0.00049, 0.00221) and paranoid ideation (*β* = 0.00066, 95% CI: 0.00013, 0.00119), increased over time, while the score for the dimension of somatization (*β* = −0.0015, 95% CI: −0.00255, −0.00045) decreased over time. The rates of increase or decrease of the scores for these three dimensions did not change significantly after 20 February.

In Wuhan users, eight out of the nine SCL-90 dimensions’ estimated scores increased faster in men than in women, except for the hostility dimension. Among the non-Wuhan users, the sex difference of the change rates was not as evident as among the Wuhan users, where three dimensions’ scores—somatization, psychoticism and hostility—increased faster in men than in women, while no sex difference was found in the other dimensions. The fixed effect models could not calculate a regression coefficient for the main effect of sex since it was a time-invariant variable. Hence, we could not compare the levels of mental health between men and women.

## 4. Discussion

There were three major findings in this study. First, the daily reported number of cumulative cases was a significant risk factor for mental health among both the Wuhan and non-Wuhan users. Although it had been reported that excessive attention to COVID-19 news (e.g., daily new case numbers) was a risk factor for mental health [25], previous studies did not quantify how people’s mental health levels changed with the accumulation of the cases.

Second, the results of the fixed effect models suggested that for non-Wuhan users, the level of mental health problems measured by all nine SCL-90 dimensions significantly increased with the duration of the epidemic. In comparison, for the Wuhan users, the impact of time on the estimated mental health levels was not that evident. Previous studies compared the mental health of people across areas at one point in time [26], but they rarely investigated the dynamic changes of people’s mental health over time. To the best of our knowledge, this is the first study to quantify the relation between the lasting time of the COVID-19 epidemic and mental health, and the results showed that the mental health of people in and out of the area of the first reported cases changed in different patterns.

Third, descriptive analysis showed that from 28 January 2020 to 20 February 2020 (the turning point of the epidemic), the estimated daily scores for the nine dimensions of SCL-90 were generally lower among the Wuhan users than the non-Wuhan users. From 20 February to 7 March, the curves for the estimated SCL-90 scores among Wuhan and non-Wuhan users were getting closer to each other. For some of the dimensions, Wuhan users’ scores reached above the non-Wuhan users on some days. To the best of our knowledge, this is the first study to report how the comparisons of estimated levels of mental health between people living in and out of the area of the first reported cases changed over time.

In the current study, we analyzed Sina Weibo data posted between 28 January 2020 and 7 March 2020 to estimate the SCL-90 mental health among social media users living in Wuhan and non-Wuhan areas. As we know, Wuhan was locked down on 23 January 2020. This is a very strict epidemic prevention measure. At the early stage of the city’s lockdown, Wuhan residents had to adjust their daily living activities for hygiene, self-quarantine requirements and stopping the use of public transportation. A substantial number of laborers who engaged in the informal economy lost their jobs. As the cases soared, more residents found that their relatives, friends and probably they themselves were infected by COVID-19, then they had to struggle to find hospital beds or masks due to shortage of health care resources [27]. At this stage, Wuhan residents were focusing on surviving or basic living needs in the challenging environment, and they might have had limited cognitive resources [28] left to perceive psychological problems. Consequently, the Wuhan users expressed lower levels of anxiety and concerns on social media in comparison with the Non-Wuhan users. The estimated daily scores for mental health in this stage (from 28 January to 20 February, the turning point of the epidemic) were generally lower among the Wuhan users than the non-Wuhan users.

In addition to the resource limitation theory [28] that we mentioned above, the phenomenon might be explained by the amplified risk perception [29] among non-Wuhan users as well as cognitive dissonance [30] among the Wuhan users. During the early stages of the outbreak, people were not only threatened by the deadly virus but also overwhelmed by rumors and misinformation [31] that led to amplified risk perception among non-Wuhan users, while people in Wuhan had direct experience and information. They could automatically adjust the “magnified” information and had a more objective understanding of the risks rather than excessive negative perceptions. Wuhan was the area most severely affected by the COVID-19 outbreak in China. People in Wuhan were unable to travel or relocate to other areas due to the lockdown policy. In order to relieve discomfort caused by the deadly COVID-19 virus, the risk perception of the people of Wuhan might have been altered by the cognitive dissonance [30] that manifested by perceiving the virus as less dangerous.

Consistent with our findings, it was reported that Chinese people living in the risk center experienced lower levels of anxiety and concerns in comparison with people living outside the risk center during the COVID-19 outbreak [32] and previous disasters (e.g., SARS [12] and the Wenchuan earthquake [33]). The phenomenon was termed the typhoon eye effect [34], which is contrary to the dominant theory of risk perception [35].

Although Wuhan was not well prepared for the attack from COVID-19 in the early stages, more resources rapidly arrived in Wuhan. 20 February was the turning point of the epidemic in China, and from then on, the epidemic was mitigated. At this stage, Wuhan residents’ attention might have moved to their own psychological concerns. We observed that the curves for the estimated SCL-90 scores among the Wuhan and non-Wuhan users were getting closer to each other. For some of the dimensions, the Wuhan users’ scores had reached above the non-Wuhan users on some days.

In the current study, we observed that the estimated SCL-90 scores were changing over time, as were the comparisons of the scores between the Wuhan and non-Wuhan users. During the 40 days of our study period, we observed that the scores of the Wuhan users were lower in the early stages but reached above those of the non-Wuhan users in some days in the late stage. This indicates that the psychological typhoon eye theory has boundaries. One previous study reported that the typhoon eye effect was observed among young adults instead of adults older than 50, among singles or among those married with one child, instead of those divorced or widowed [36]. Another study observed an inverted U-shaped relationship between the geographical distance to Wuhan and burn out among working adults [37]. All this evidence suggests that the psychological typhoon eye theory should be interpreted with caution.

In this study, we found that people’s mental health deteriorated with the rapid accumulation of COVID-19 cases. As the outbreak accelerated, daily updates of the cumulative number of cases, number of new cases and death toll attracted great attention from the public. When facing this information, people were assessing the risk of infection and the harm of COVID-19 [38]. People might have perceived COVID-19 as a tremendous threat when the cumulative number of cases increased rapidly, and consequently, they might have experienced cognitive biases [39] that manifested as exaggerated perceived risk and excessive panic [40]. During the outbreak, some people doubted that they had contracted COVID-19 due to a few coughs or after an outdoor walk. Then, they searched through a large amount of information to judge whether they did have COVID-19. This might have led to information overload and, consequently, negative emotions [26].

A long-lasting negative state may cause deterioration of people’s mental health. Our longitudinal analysis suggested that people’s mental health was not only affected by the cumulative number of cases that marked the severity of the epidemic, but it was also significantly affected by the length of time that the epidemic lasted. We found that among the non-Wuhan users, all nine dimensions of the estimated SCL-90 scores increased over time. Among the Wuhan users, the dimensions’ scores for psychoticism and paranoid ideation increased, while the somatization dimension score decreased over time, indicating that the psychoticism and paranoid ideation symptoms increased over time, whereas the somatization symptoms decreased over time.

A plausible explanation for the reduction of somatization symptoms over time might be stress-induced analgesia [41]. Pain is the major symptom measured by the SCL-90 somatization dimension. A large number of studies reported the phenomenon of pain suppression upon exposure to stressful or fearful stimuli in humans. During the current study period (From January 2020 to March 2020), Wuhan residents had to face the life-threatening virus in a locked down city. Such a frightening experience may activate the inhibitory pain pathway through a large number of neurotransmitters and neuropeptides [41], and consequently, Wuhan residents’ somatization symptoms might reduce over time. However, this was not necessarily concurrent with an improvement in mental health.

To the best of our knowledge, this is the first study to employ a longitudinal study design with daily estimates of a population’s mental health status during the COVID-19 pandemic. It is generally not feasible to ask study participants to answer the same set of questionnaires every day. Artificial intelligence makes the daily estimation of one’s mental health status possible. We utilized a machine learning algorithm to calculate the daily SCL-90 scores according to the online information that the study participants left on social media. Hence, the current study obtained 40 days of daily estimations of SCL-90 scores. This novel method provided us opportunities to monitor people’s mental health in a timely manner during the epidemic and subsequently guide the production of relevant coping strategies.

The study results should be interpreted in the context of the study’s limitations. The model-based estimation of the SCL-90 scores was not the same as the self-reported SCL-90 scores, though they were moderately correlated. The location information was self-reported by Weibo users when they registered on the Sina Weibo platform. For most social media users, the registered address is either their home or their long-term workplace. Nevertheless, there might be a few users who lived in another city instead of their registered location on Weibo during the COVID-19 outbreak. However, we considered that they were only a small portion of the study sample, since the study period was right after the lockdown of Wuhan, and generally, traveling in or out of Wuhan was impossible at that time. We considered that the potential slight misclassification of the study sample with regard to their locations might have little impact on the study results. The ages of three quarters of the study participants were unknown, and among those who reported their ages, the majority were young adults who were active in their online communities. They could not represent the general Chinese population. Future studies which compare the mental health of young Weibo users and a representative sample of the Chinese population would be interesting.

Although the estimated scores for several dimensions of the SCL-90 were lower among the Wuhan users than the non-Wuhan users, we do not believe that the mental health of Wuhan users was better than the non-Wuhan users. A plausible explanation is that Wuhan users were focusing on fighting the virus and surviving in the challenging environment, and they might not have realized their psychological concerns until the epidemic slowed down in Wuhan. It is necessary to conduct future studies to investigate the mental health of Wuhan residents after the city lifted its lockdown.

## 5. Conclusions

People’s mental health deteriorated with the acceleration of the COVID-19 epidemic, due to both the rapid accumulation of cases and the lengthy duration of the epidemic significantly impacting people’s mental health. The aspects and magnitude of the changes in mental health among the Wuhan and non-Wuhan users were different. During the study period, the Wuhan users expressed less psychological concerns in comparison with the non-Wuhan users. Controlling the outbreak as soon as possible is the key to relieving people’s mental health problems. Meanwhile, we should develop and tailor strategies to address the different mental health issues among people living in and out of the area where the first cases were reported.

## Figures and Tables

**Table 1:** Inclusion and exclusion criteria for the study sample.

Types	Inclusion Criteria	Exclusion Criteria
Number of Weibo posts	At least 50	Less than 50
Authentication type	Individual user	Institution user
Region	Mainland China	Overseas, other

**Table 2:** Repeated measures ANOVA to compare mental health among the Wuhan and non-Wuhan residents.

	28 January 2020		7 February 2020		17 February 2020		27 February 2020		7 February 2020		Repeated Measures	
	Mean	Std	Mean	Std	Mean	Std	Mean	Std	Mean	Std	ANOVA	
											*F* Value	*p* Value
Somatization												
Wuhan	1.59	0.31	1.61	0.29	1.68	0.4	1.66	0.37	1.65	0.3		
Non-Wuhan	1.59	0.26	1.65	0.3	1.69	0.32	1.67	0.31	1.66	0.3	2.04	0.1535
Anxiety												
Wuhan	1.74	0.36	1.71	0.29	1.78	0.37	1.78	0.35	1.79	0.32		
Non-Wuhan	1.77	0.32	1.77	0.36	1.82	0.38	1.81	0.37	1.8	0.36	5.13	0.0235
Obsessive-Compulsive Disorder												
Wuhan	1.88	0.25	1.87	0.3	1.94	0.37	1.91	0.31	1.93	0.31		
Non-Wuhan	1.88	0.24	1.89	0.28	1.94	0.31	1.92	0.3	1.92	0.28	0.46	0.4987
Interpersonal Sensitivity												
Wuhan	1.69	0.19	1.67	0.21	1.71	0.22	1.7	0.21	1.72	0.22		
Non-Wuhan	1.72	0.22	1.69	0.24	1.73	0.25	1.73	0.25	1.72	0.26	6.31	0.012
Depression												
Wuhan	1.99	0.36	1.98	0.36	2.01	0.39	1.99	0.36	2.02	0.35		
Non-Wuhan	1.98	0.35	2	0.4	2.05	0.4	2.03	0.4	2.02	0.4	3.4	0.0654
Psychoticism												
Wuhan	1.65	0.28	1.65	0.3	1.69	0.34	1.67	0.32	1.71	0.31		
Non-Wuhan	1.67	0.24	1.67	0.27	1.7	0.29	1.69	0.29	1.7	0.28	2.11	0.146
Paranoid Ideation												
Wuhan	0.93	0.29	0.95	0.28	1.02	0.5	1	0.3	1.04	0.32		
Non-Wuhan	0.99	0.33	0.99	0.36	1.05	0.4	1.05	0.4	1.04	0.42	9	0.0027
Hostility												
Wuhan	1.41	0.16	1.41	0.16	1.44	0.24	1.44	0.17	1.43	0.18		
Non-Wuhan	1.43	0.18	1.43	0.19	1.45	0.21	1.45	0.21	1.44	0.2	3.94	0.0473
Phobic Anxiety												
Wuhan	1.33	0.13	1.32	0.13	1.35	0.23	1.34	0.2	1.34	0.16		
Non-Wuhan	1.34	0.16	1.34	0.18	1.36	0.2	1.35	0.19	1.34	0.19	4.6	0.032

Note: From 28 January to 7 March, computerized models estimated the daily SCL-90 scores for each individual for 40 days. Repeated measures ANOVA utilized the 40 days’ estimated scores to compare the mental health levels between Wuhan and non-Wuhan residents. The above table only lists the scores for 5 days.

**Table 3:** Comparison of SCL-90 scores in the COVID-19 period with the Chinese norm.

	All Time		Population Norm		t	*p*
	(*n* = 16,453)		(*n* = 1388)			
	Mean	Std	Mean	Std		
Somatization						
Wuhan	1.64	0.03	1.37	0.48	69.45	<0.0001
Non-Wuhan	1.66	0.02	1.37	0.48	75.08	<0.0001
Anxiety						
Wuhan	1.75	0.03	1.39	0.43	105.61	<0.0001
Non-Wuhan	1.79	0.02	1.39	0.43	118.09	<0.0001
Obsessive-Compulsive Disorder						
Wuhan	1.9	0.03	1.62	0.58	61.65	<0.0001
Non-Wuhan	1.91	0.02	1.62	0.58	63.97	<0.0001
Interpersonal Sensitivity						
Wuhan	1.69	0.02	1.65	0.61	8.57	<0.0001
Non-Wuhan	1.72	0.01	1.65	0.61	13.85	<0.0001
Depression						
Wuhan	1.99	0.02	1.5	0.59	104.54	<0.0001
Non-Wuhan	2.02	0.02	1.5	0.59	112.03	<0.0001
Psychoticism						
Wuhan	1.67	0.02	1.29	0.42	112.74	<0.0001
Non-Wuhan	1.68	0.02	1.29	0.42	119.21	<0.0001
Paranoid Ideation						
Wuhan	1.39	0.01	1.43	0.57	−8.99	<0.0001
Non-Wuhan	1.41	0.01	1.43	0.57	−4.49	<0.0001
Hostility						
Wuhan	1.4	0.02	1.46	0.55	−13.89	<0.0001
Non-Wuhan	1.41	0.01	1.46	0.55	−11.64	<0.0001
Phobic Anxiety						
Wuhan	1.33	0.01	1.22	0.41	34.59	<0.0001
Non-Wuhan	1.35	0.01	1.22	0.41	39.07	<0.0001

**Table 4:** Results of the fixed effect models, analyzing the effect of the cumulative case number and lasting time of the epidemic on the SCL-90 mental health among Wuhan and non-Wuhan users.

	Date				Cumulative Cases (Every 1000 Cases)			
	β	SE	95% CI	*p* Value	β	SE	95% CI	*p* Value
Somatization								
Wuhan	−0.0015	0.00054	(−0.00255, −0.00045)	0.005	0.00238	0.00028	(0.00183, 0.00293)	<0.0001
Non-Wuhan	0.00185	0.00005	(0.00175, 0.00195)	<0.0001	0.08152	0.00541	(0.07092, 0.09212)	<0.0001
Anxiety								
Wuhan	−0.00031	0.0005	(−0.00129, 0.00067)	0.5357	0.00165	0.00026	(0.00114, 0.00216)	<0.0001
Non-Wuhan	0.00217	0.00005	(0.00207, 0.00227)	<0.0001	0.03419	0.00589	(0.02265, 0.04573)	<0.0001
Obsessive-Compulsive Disorder								
Wuhan	−0.00029	0.00048	(−0.00123, 0.00065)	0.5434	0.00179	0.00025	(0.0013, 0.00228)	<0.0001
Non-Wuhan	0.0017	0.00004	(0.00162, 0.00178)	<0.0001	0.04931	0.00522	(0.03908, 0.05954)	<0.0001
Interpersonal Sensitivity								
Wuhan	0.00006	0.00039	(−0.0007, 0.00082)	0.8726	0.00092	0.0002	(0.00053, 0.00131)	<0.0001
Non-Wuhan	0.00065	0.00004	(0.00057, 0.00073)	<0.0001	0.00187	0.00461	(−0.00717, 0.01091)	0.6843
Depression								
Wuhan	0.00089	0.00061	(−0.00031, 0.00209)	0.1441	0.00103	0.00032	(0.0004, 0.00166)	0.0012
Non-Wuhan	0.002	0.00006	(0.00188, 0.00212)	<0.0001	0.04157	0.00718	(0.0275, 0.05564)	<0.0001
Psychoticism								
Wuhan	0.00135	0.00044	(0.00049, 0.00221)	0.002	0.00043	0.00023	(−0.0002, 0.00088)	0.0572
Non-Wuhan	0.00152	0.00004	(0.00144, 0.0016)	<0.0001	0.01603	0.0049	(0.00643, 0.02563)	0.0011
Paranoid Ideation								
Wuhan	0.00066	0.00027	(0.00013, 0.00119)	0.0155	0.0005	0.00014	(0.00023, 0.00077)	0.0003
Non-Wuhan	0.00076	0.00002	(0.00072, 0.00080)	<0.0001	0.02081	0.00282	(0.01528, 0.02634)	<0.0001
Hostility								
Wuhan	−0.00025	0.0004	(−0.00098, 0.00048)	0.5	0.00108	0.00019	(0.00071, 0.00146)	<0.0001
Non-Wuhan	0.00067	0.00003	(0.00061, 0.00073)	<0.0001	0.02336	0.00363	(0.01625, 0.03047)	<0.0001
Phobic Anxiety								
Wuhan	−0.0083	0.00514	(−0.01837, 0.00177)	0.1064	0.01431	0.00267	(0.00908, 0.01954)	<0.0001
Non-Wuhan	0.00051	0.00003	(0.00045, 0.00057)	<0.0001	0.016	0.0038	(0.00855, 0.02345)	<0.0001
	Date*Turning Point				Date*Sex			
	β	SE	95% CI	*p* value	β	SE	95% CI	*p* value
	(×10^−5^)	(×10^−5^)	(×10^−5^)		(×10^−5^)	(×10^−5^)	(×10^−5^)	
Somatization								
Wuhan	−0.02534	0.02986	(−0.08387, 0.03319)	0.3961	−0.75231	0.25585	(−1.25378, −0.25084)	0.0033
Non-Wuhan	−0.09667	0.005	(−0.10647, −0.08687)	<0.0001	−0.08579	0.04173	(−0.16758, −0.00400)	0.0398
Anxiety								
Wuhan	0.0103	0.02774	(−0.04407, 0.06467)	0.7103	−0.9901	0.23761	(−1.45582, −0.52438)	<0.0001
Non-Wuhan	−0.06934	0.00493	(−0.07900, −0.05968)	<0.0001	−0.08011	0.04541	(−0.16911, 0.00889)	0.0777
Obsessive-Compulsive Disorder								
Wuhan	−0.05847	0.02661	(−0.11063, −0.00631)	0.028	−0.94765	0.22799	(−1.39451, −0.50079)	<0.0001
Non-Wuhan	−0.06839	0.00437	(−0.07696, −0.05982)	<0.0001	−0.01474	0.04022	(−0.09357, 0.06409)	0.714
Interpersonal Sensitivity								
Wuhan	0.01873	0.02194	(−0.02427, 0.06173)	<0.0001	−0.73278	0.18792	(−1.10110, −0.36446)	<0.0001
Non-Wuhan	0.03987	0.00386	(0.03230, 0.04744)	<0.0001	0.05096	0.03553	(−0.01868, 0.12060)	0.1515
Depression								
Wuhan	−0.10777	0.03387	(−0.17416, −0.04138)	0.0015	−1.62816	0.29015	(−2.19685, −1.05947)	<0.0001
Non-Wuhan	−0.10464	0.00601	(−0.11642, −0.09286)	<0.0001	−0.03297	0.0553	(−0.14136, 0.07542)	0.5511
Psychoticism								
Wuhan	−0.04575	0.02431	(−0.09340, 0.00190)	0.0599	−0.77447	0.20829	(−1.18272, −0.36622)	0.0002
Non-Wuhan	−0.03743	0.0041	(−0.04547, −0.02939)	<0.0001	−0.11232	0.03777	(−0.18635, −0.03829)	0.0029
Paranoid Ideation								
Wuhan	−0.02545	0.01509	(−0.05503, 0.00413)	0.0917	−0.48239	0.12928	(−0.73578, −0.22900)	0.0002
Non-Wuhan	−0.00617	0.00236	(−0.01080, −0.00154)	0.0089	−0.03463	0.02171	(−0.07718, 0.00792)	0.1107
Hostility								
Wuhan	0.00004	0.02066	(−0.04046, 0.04054)	0.9985	0.01179	0.17701	(−0.33515, 0.35872)	0.9469
Non-Wuhan	−0.00886	0.00304	(−0.01482, −0.00290)	0.0036	−0.06978	0.02798	(−0.12462, −0.01494)	0.0126
Phobic Anxiety								
Wuhan	−0.66251	0.28647	(−1.22399, −0.10103)	0.0208	−5.22384	2.45415	(−10.03397, −0.41371)	0.0333
Non-Wuhan	−0.03935	0.00318	(−0.04558, −0.03312)	<0.0001	−0.01553	0.02927	(−0.07290, 0.04184)	0.5956

Note 1: dependent variable = estimate scores for the 9 dimensions of SCL-90; independent variables = date, cumulative cases (thousands) and two interaction terms: date*turning point and date*sex. Note 2: most of the numbers in this table were rounded to 5 decimal places, and since some of the numbers were very small, they became 0, if only keep two decimal places.

## Data Availability

The data presented in this study are available on request from the corresponding author.
